# First steps towards the detection of contact layers in Bangime: a multi-disciplinary, computer-assisted approach

**DOI:** 10.12688/openreseurope.14339.1

**Published:** 2022-01-21

**Authors:** Abbie Hantgan, Hiba Babiker, Johann-Mattis List

**Affiliations:** 1 Langage, Langues et Cultures d'Afrique (LLACAN, UMR 8135), Centre national de la recherche scientifique (CNRS) and l’Institut National des Langues et Civilisations Orientales (INALCO), Paris, France; 2 Department of Linguistic and Cultural Evolution, Max Planck Institute for the Science of Human History, Jena, Germany; 3 Department of Linguistic and Cultural Evolution, Max Planck Institute for Evolutionary Anthropology, Leipzig, Germany

**Keywords:** language isolates, West Africa, homozygosity, computer-assisted language comparison, language contact

## Abstract

Bangime is a language isolate, which has not been proven to be genealogically related to any other language family, spoken in Central-Eastern Mali. Its speakers, the Bangande, claim affiliation with the Dogon languages and speakers that surround them throughout a cliff range known as the Bandiagara Escarpment.  However, recent genetic research has shown that the Bangande are genetically distant from the Dogon and other groups. Furthermore, the Bangande people represent a genetic isolate.  Despite the geographic isolation of the Bangande people, evidence of language contact is apparent in the Bangime language. We find a plethora of shared vocabulary with neighboring Atlantic, Dogon, Mande, and Songhai language groups. To address the problem of when and whence this vocabulary emerged in the language, we use a computer-assisted, multidisciplinary approach to investigate layers of contact and inheritance in Bangime. We start from an automated comparison of lexical data from languages belonging to different language families in order to obtain a first account on potential borrowing candidates in our sample. In a second step, we use specific interfaces to refine and correct the computational findings. The revised sample is then investigated quantitatively and qualitatively by focusing on vocabularies shared exclusively between specific languages. We couch our results within archeological and historical research from Central-Eastern Mali more generally and propose a scenario in which the Bangande formed part of the expansive Mali Empire that encompassed most of West Africa from the 13 ^th^ to the 16 ^th^ centuries. We consider our methods to represent a novel approach to the investigation of a language and population isolate from multiple perspectives using innovative computer-assisted technologies.

## Plain language summary

Bangime is a language isolate spoken among the Dogon, Mande, Atlantic, and Songhai language families in Central-Eastern Mali. Despite the Dogon disapproval, the speakers of the Bangime “Bangande” claim an ethnic identity with the Dogon. The Bangande are geographically isolated and current genetic research denoted their genetic uniqueness. However, here we show evidence of shared vocabulary among the Bangime and neighboring language groups. We investigate the layers of contact using a computer-assisted, multidisciplinary approach in a series of steps. We use lexical automated comparisons taking into account the qualitative and quantitative measures and the correction of the findings. Within archeological and historical contexts from Central-Eastern Mali, our results show that the Bangime language was spoken before the Dogon Expansion in the Escarpment 1400c. AD. This work represents a great mark in computational linguistic for the study of language isolates and the paradox of their history.

## 1 Introduction

**Figure 1.**
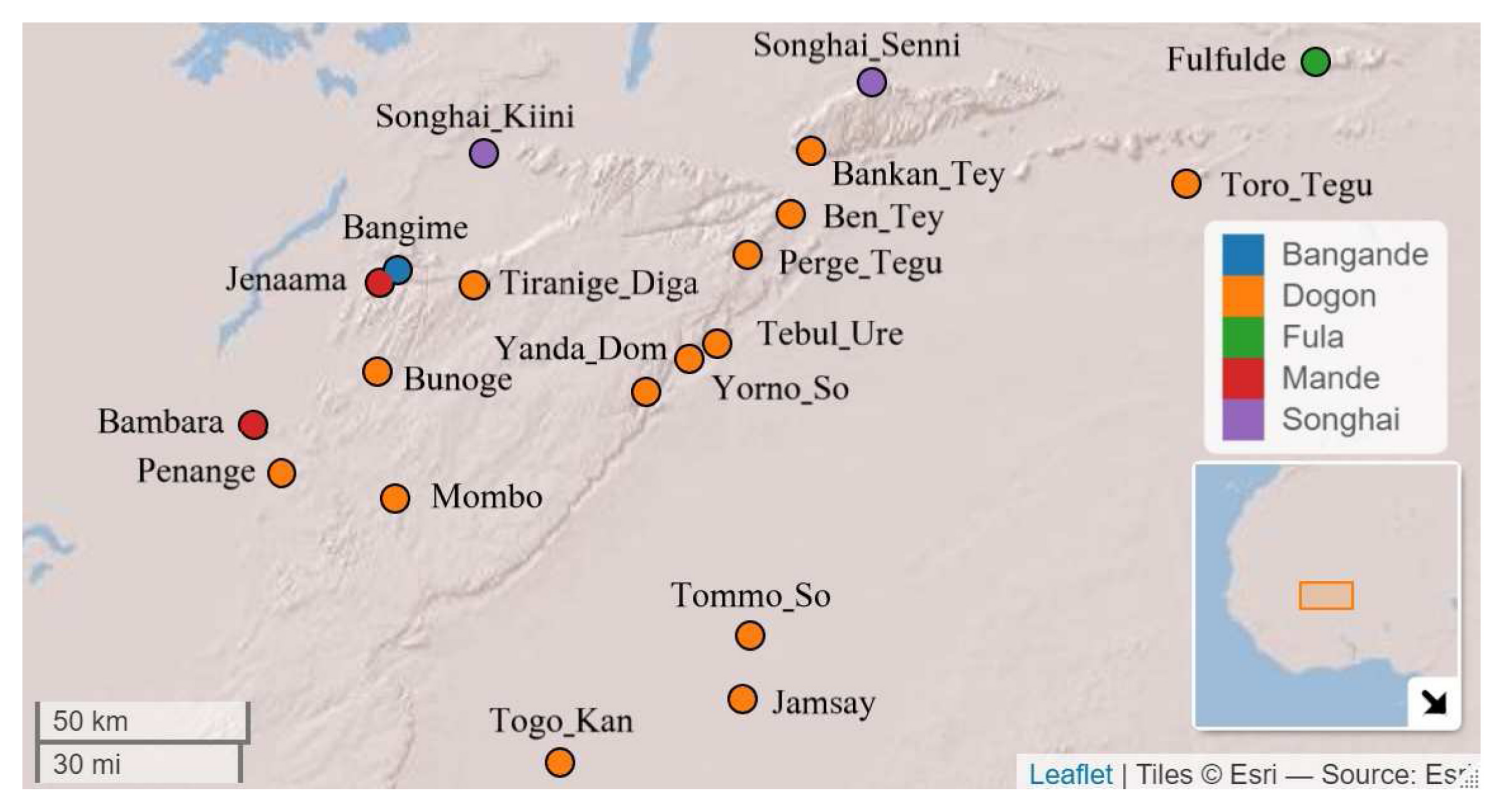
Languages used in the data sample. Map created with LingTypology R-Package ( Moroz, 2017). Coordinates and classification from Glottolog (Glottolog, https://glottolog.org, Hammarström *et al.*, 2021) and Dogon and Bangime Linguistics Project ( http://dogonlanguages.org).

Bangime, a language isolate spoken in central-eastern Mali, represents an enigma, not only in terms of linguistics, but also with regards to past ethnographic affiliations and migration patterns. The speakers of Bangime, the Bangande, live among and claim to constitute one of the Dogon groups that also occupy the rocky terrain of the Bandiagara Escarpment. However, there is little evidence in support of the Bangande being genetically affiliated with the Dogon or speaking one of the estimated 21 Dogon languages, nor of their being related to the neighboring Mande-speaking groups who inhabit a valley which stretches from the west and ends at the eastern edge of the Escarpment. Further to the north of the area where Bangime is spoken lies the vast Sahara Desert, the southern borders of which are occupied by Songhai-speaking populations. Throughout the region are found Fula semi-nomadic herders who speak Fulfulde. Thus, we know that the Bangande have had the opportunity to engage in contact with each of these populations, but because there are no written historical records of their past settlement and migration patterns, nor have there been any archeological investigations of the western portions of the Bandiagara Escarpment where the Bangande are found today, we must rely on data from the present to reconstruct a picture of the past. Figure 1 illustrates the geographic positions of the languages represented in the sample with respect to where Bangime is spoken. Note that the points represent approximations; languages such as Fulfulde have a reach throughout the entire region and even beyond to bordering nations.

Although multidisciplinary data are available concerning Dogon, Fula, Mande, and Songhai social history and material culture, the precise genealogical positioning of their languages within a larger context remains a mystery. Since Bangime represents a puzzle in this mosaic picture of central-eastern Malian languages, a closer understanding of its previous contacts will help to shed light on the deeper relations to these bordering languages. For the purposes of the current study, we focus on the linguistic (lexical) and genetic (genome-wide genotyping) data from a sample of these five ethno-linguistic groups and couch our findings in the historical background of what is known about these groups. Naturally, the area which is currently inhabited by the Bangande and neighboring populations was occupied by peoples of potentially pre-ethnic delineations; we leave this matter to future research.

We present a computer-assisted, multidisciplinary, first approach to addressing this problem of detecting the layers of contact in Bangime. First, we assemble lexical evidence of contact between Bangime speakers with their neighboring languages, using a computer-assisted technique, followed by an evaluation of the materials by contrasting them with genetic findings. Specifically, we propose trajectories for Bangande settlement patterns. With this study, we lay the foundation of future collaborative work that will improve, correct, and enhance the results of this study. The original data used for the study are made available so that additional researchers may follow up on, and test our hypotheses concerning contact layers in Bangime.

### 1.1 Current ethno-linguistic situation

In addition to Bangime, we focus on Fulfulde, plus languages from the Dogon, Mande, and Songhai affiliations for the purposes of the current study. Fulfulde, plus the Dogon and Mande, are thought to be distantly related in that each constitutes a separate branch of the Niger–Congo language phylum, while Songhai is that of Nilo–Saharan (compare Glottolog, https://glottolog.org, Hammarström *et al.*, 2021; Ethnologue, 2021). Bangime, as noted above, is a language isolate, considered by some researchers to be one of only four confirmed isolates spoken on the African continent ( Blench, 2017: 167).

Based on clay pot creation techniques, Mayor *et al*. (2005) consider Bozo fishermen of the Mande ethnolinguistic group to be the original inhabitants of the area directly to the west of the Bangime-speaking villages. However, oral traditions recount warfare between the Bangande and Bozo groups and both claim first-comer status. Although the Swiss-based archeological group spent a significant amount of time examining the Bandiagara Escarpment, their studies focused on the central and eastern portions of the cliff range rather than the area where Bangime and the southwestern Dogon languages are currently spoken ( Mayor *et al.*, 2005; Mayor *et al.*, 2014; Mayor *et al.*, 2016). In those areas, they have found evidence for Dogon inhabitation of the area dating back to between the 13 ^th^ and the 15 ^th^ centuries AD. Otherwise, both the Dogon and Bangande oral histories tell of Fula slave invaders who probably impacted the region from the 17 ^th^ to the 19 ^th^ centuries AD ( Fay, 1997). The Songhai likely had more of a peripheral influence on the Bangande; their empire is discussed in Section 2 below.

Therefore, we do not have the option to rely on either historical records or on the use of cognates between Bangime and the surrounding languages, but we can examine the outputs of automatically detected borrowings. To this end, we can seek to answer the question: How does a language isolate retain lexical (and grammatical) borrowings/does it do so in a manner that differs from languages that are part of a group/clade?

### 1.2 Current genetic relationships

Here, we report results from current research which has revealed patterns of genetic structure and admixture among the populations of central–eastern Mali ( Babiker *et al*., 2020). This work utilized genetic data to reveal the mystery of the language isolate Bangime and its genetic relationship with neighboring populations. The study shows that the genetic and linguistic patterns of the Bangime language and its speakers are in agreement. In detail, Babiker *et al.* (2020) show that the Bangande population is a genetic isolate that has resisted assimilation and language replacement maintained by geographical isolation.

The results show that populations of central–eastern Mali are of West African origin and closely related to non-Bantu speaking populations of the Niger–Congo language superfamily. Further, the Bangande showed the highest excess homozygosis in comparison to Dogon and other groups pointing to the impact of genetic drift on populations with small effective population size and consanguinity practices.

Furthermore, the modern-day Dogon populations display little or no admixture from Africans and non-Africans. Instead, they show signals of relative isolation and homogeneity suggesting that the ancestors of these populations inhabited the region prior to later waves of migrations and that the geographical isolation of these groups across the Bandiagara Escarpment and the Dogon Plateau might have served as a genetic barrier to gene flow.

### 1.3 Current hypotheses and challenges

***1.3.1 Current hypotheses.*** It appears that the Bandiagara region was settled in waves (and this is mentioned specifically by art historian Huib Blom (2011: 18). The Mande expansion was much larger than that of the Dogon, and thus their genetic and linguistic diversity reflects that magnitude: the Dogon are a much more closely knit population and group of languages.

The linguistic and genetic structure patterns of the Bangande population suggest that it might represent the first wave of migration that settled in the Escarpment and resisted both gene flow and language replacement across the generations. In contrast, the linguistic and genetic structure patterns of the Dogon hint to a later wave that resulted in the expansion of the Dogon farmers and the diversification of the Dogon languages in the last ~1,000–3,000 years.

***1.3.2 Current challenges.*** The comparative study of an isolate is challenging for many reasons. Primarily concerning Bangime as a language isolate, linguistic data for Bangime and other languages are available, but since Bangime cannot be genealogically affiliated with any other language, it is difficult to draw conclusions from its comparison with other languages. To compensate for this difficulty, apart from sharing inherited traits, languages also exchange material through contact, so the contact relations with Bangime can be inferred by consistently looking into this evidence, and comparing those patterns of contact which can be identified to try to make a coherent image out of the puzzle of pieces. The challenge here, however, is to identify the coherent contact layers: when languages migrate along with their speakers, and throughout their history, they will take material in different times and contact situations. These can be identified as contact layers, but so far, there is no coherent method for the identification of contact layers. Thus, scholars have usually used multiple pieces of evidence such as external historical evidence, internal semantic evidence, sound correspondences (though these are often the hardest to analyze), and external evidence from other disciplines, *e.g.*, archaeogenetics, or genetics, as these can confirm scenarios of contact among speakers of a population.

In this study, we show how linguistic evidence from contact relations can be assembled in a coherent and transparent way, thus open for criticism and expansion through later work. Given the necessity to make use of external evidence when investigating contact layers, we illustrate how preliminary collaboration and comparison with genetic findings can help us to shed light on the history of the Bangime language and its speakers. Our research contributes to these historical hypotheses with data from both DNA and languages, analyzed with ground-breaking comparative computational methods.

## 2 Background

Besides oral histories, practically nothing is known about the past of the Bangande. The first time the Bangime language was mentioned in the literature was in the 1950s ( Bertho, 1953; Calame-Griaule, 1956), describing the fact that Bangime is markedly different from Mande or Fula, as well as from the Dogon languages. Blench (2005: 15–16); Blench (2007: 3) was the first to state that Bangime is an isolate. For the other groups in our sample, we summarize the most pertinent details to our study in chronological order to their estimated arrival in the area as follows.

### 2.1 Mande

Beyond Bantu, Mande peoples and languages represent a relatively neglected part of West African ethnography; the term “Mande Expansion” was first used by Brooks (1993) in reference to the progressively southward movement of Mande-speaking warriors from the increasingly aridifying Sahara from 1100–1500 AD. Furthermore, Brooks states that the raiders were but the second of two waves of Mande peoples in which the former constituted trading peoples along the trans-Saharan routes from the far north to the central West African coast. The ripples of the Mande Expansion are felt that much more so in the present. Today, according to Glottolog, the Mande ethno-linguistic group consists of 75 languages and 172 dialects ( Hammarström *et al.*, 2021) spoken by upwards of 30 to 40 million people ( Vydrin, 2009). The reason for this vast and far-reaching, yet recent, expansion lies in the people’s presence within both the Mali (13–15 ^th^ C. AD) as well as the Ghana (8–12 ^th^ C. AD) Empires. Among our Mande groups, Bambara, Bozo, and Soninke, the Bozo population in this study is not necessarily representative of the main Bozo groups of fishing villages along the Niger River and its floodplains. The “cliffs Bozo” we sampled here are suspected to represent linguistically converted Bozo speakers, perhaps originally speakers of a Soninke-like language (Jeffrey Heath, unpublished study).

### 2.2 Songhai

Although an impact from Songhai peoples and languages including the Dia Dynasty which was likely composed of a mix of Songhai and other groups ( Arazi, 2005) and existed much earlier, the Songhai Empire took place from the 15–16 ^th^ C. AD ( Brooks, 1985). Our samples from the Kikara, Tondi Songwai Kiini (hereafter simply ‘Kiini’), and Humbori, Humburi Senni Songhai (which we refer to as ‘Senni’), villages belong to the eastern division of Songhai languages spoken in Mali, which have been classified within the Nilo-Saharan language superfamily. Tondi Songwai Kiini spoken in Kikara is distinguished from other Songhai languages as the “mountain Songhay language” ( Heath, 2005a).

### 2.3 Dogon

As noted above, the Bangande, speakers of Bangime, claim a Dogon ancestry. The Dogon themselves have been the topic of numerous studies and surveys dating back to the early 20 ^th^ C. AD ( Desplagnes, 1907). The most up to date of these, Mayor *et al*. (2005); Mayor *et al*. (2014) and Mayor *et al*. (2016) propose a relatively recent (14 ^th^–16 ^th^ C. AD) settlement of the Bandiagara Escarpment by the Dogon peoples based on traditional funerary practices and radiocarbon dating from different sites covering past cultures. It is likely that the Dogon took refuge in the caves and cliffs of the Escarpment in order to protect themselves from imposing empires, slave raids, and religious persecution. Because of this geographic isolation, many Dogon people maintain a traditional way of life even today.

It is currently thought that the Dogon speak at least 22 distinct languages (Dogon and Bangime linguistics project, http://dogonlanguages.org). Within the variation attested among the Dogon languages, the lowest limit for mutual intelligibility based on lexical estimates is 32% ( Prokhorov *et al*., 2012).

### 2.4 Atlantic

Although it is slightly problematic for our automatic detection methods, Fulfulde is the only Atlantic language in our current sample. The reason for this is that it is only Fulfulde (Maasina dialect) speakers that have any contact with the Bangande, however in future studies it will be beneficial to include outside groups for comparison.

According to Fay (1997), the presence of Fula in the wider area has existed since the 13 ^th^ century. Mayor *et al.* (2005: 30), however, propose that the Fula did not have contact with the Dogon until the 17 ^th^ century onwards. Either Fula or Songhai peoples could have brought the initial influence of Islam to the Bandiagara Escarpment populations, but this is a recent development and not all Dogon nor Bangande practice Islam today.

### 2.5 Bangande

As only the Bangande speak their language, Bangime, they are all to some extent bilingual, but few are multilingual. The only language passed from parents to children in the seven villages where Bangime is spoken is Bangime. The primary *lingua franca* of the area is Fulfulde, which Bangime speakers use to communicate not only with Fula animal herders but also with Dogon and Mande-speakers. It is only through travel to the regional capital Mopti or the country’s capital Bamako that Bangande become conversant in Bambara. Otherwise, the only Bangande who speak languages other than Bangime are those that grew up outside of the seven Bangime-speaking villages. These include spouses (women) and migrants who moved from other, often Tommo So Dogon-speaking, villages. Specifically, blacksmithing communities, discussed below, in Bangande villages are of Tommo So heritage.

Bangande divide themselves into two categories: those of “noble” and “slave” castes. Despite the likelihood that these two classes are a superimposed relic from the Mali Empire, (see results and discussion in Section 4 and Section 5), today social hierarchy is organized according to these roles which are assigned by one’s heritage (birth). Thus, a Bangande village chief and his lineage may only marry those of the noble caste and not anyone from the slave caste. On the other hand, those of the slave caste may marry from outside the Bangande community, and thus it is possible for Dogon women to move to and integrate into one of the Bangime-speaking villages. Furthermore, it is claimed by the Bangande themselves that those of the noble caste are the only “true” Bangande whereas others, many of whose last names are associated with Dogon or Mande clans, are of a “mixed” ancestry. Therefore, persons who speak Dogon or Mande languages are considered by the Bangande to be of a “mixed” genealogical origin.

### 2.6 Caste system

In addition to the five groups considered for this study is the elaborate caste system that transcends ethno-linguistic delineations in Mali and beyond. That is, “endogamous artisan and musician groups”, as well as “noble” and “freeborn” ( Tamari, 1991: 221, 223) societies are found throughout West Africa, living separately, but along-side established ethno-linguistic groups. Based on both historical records and linguistic comparison of lexical borrowings, Tamari (1991); Tamari (1995), who specifically discusses the caste system among 15 West African groups, including the Fula, Dogon, “Manding” (including Bambara), and Soninke, proposes that all caste systems originated from either the “Manding”, Soninke, or Wolof (another Atlantic group) peoples, no later than 1500 AD. She further notes that borrowings abound throughout West Africa for terminology associated with the caste system. Intriguingly, she notes that the word for ‘noble’ which is used among the languages of West Africa is from Arabic (p. 224), and Hantgan (under review) notes that the wide-spread word for ‘ethnicity’ is also a possible loan from Arabic. Our findings touch on these issues.

## 3 Methods

All of the lexical data used for this study were gathered independently and for purposes other than comparative use; most were collected as part of dictionaries or lexicons. For this reason, in addition to procuring the data described in subsection 3.1, we had to prepare each transcription and gloss using the methods described in subsection 3.2.

### 3.1 Materials

***3.1.1 Linguistic data.*** We focus on the languages that immediately surround Bangime, and those that have potentially had a historical impact on the language through past contact with its speakers. This resulted in a total of 38 languages from 4 languages families, as shown in Table 1.

**Table 1.** Languages used in the sample geographically and genealogically grouped.

Language family	Subgroup	Varieties
Dogon	Northwestern	Bondu So, Dogul Dom, Tebul Ure, Yanda Dom, Yorno So
	Northeastern	Bankan Tey, Ben Tey, Jamsay, Gourou, Perge Tegu, Nanga, Toro Tegu
	Southeastern	Donno So, Togo Kan, Tommo So, Tomo Kan, Toro So
	Southwestern	Ampari, Bunoge, Mombo, Penange, Tiranige Diga
Mande	Western	Bambara
	Soninke-Bozo	Bozo Debo, Bozo Hainyaxo, Bozo Korondougou nord, Bozo Korondougou sud, Bozo Kotya, Bozo Pondori nord, Bozo Pondori sud, Bozo Tieyaxo, Bozo Tiema Cewe, Jenaama Bozo, Soninke
Atlantic	Northcentral Atlantic	Fulfulde
Nilo-Saharan	Eastern Songhai	Kiini, Senni

The sources for the data are as follows: Dogon data come from the Dogon Languages Project lexical database ( Heath *et al.,* 2015). The subgroupings for the Dogon languages are based on an on-going phylogenetic study of the Dogon languages ( Hantgan, 2019). Bangime data are from Heath *et al.* (2019). Those from Songhai Kiini and Senni are drawn from Heath (2005b) and Heath (2015) respectively. Jenaama data are from Heath (2016). Remaining language data are from the pan-African lexical database RefLex ( http://reflex.cnrs.fr, Segerer & Flavier, 2011–2022), (with specific source information given in the supplemental materials), and the first author’s knowledge of Fulfulde and Bambara. Mande and Atlantic classifications are based on Glottolog ( Hammarström *et al*., 2021).

There are 38 languages represented in the sample of Malian languages used for this study. The number of concepts selected was 348. As some languages have more than one form, and others are missing certain concepts, the total number of words in the lexical dataset is 9577. From a statistical standpoint, a limitation of this dataset is its somewhat skewed coverage. Despite this, we decided not to remove languages or concepts as was done in Hantgan and List (personal communication), because of the qualitative aspect of the study; it was crucial for us to examine individual lexical items in the borrowing context despite the fact that they were not represented across all languages so as to at least find tendencies and directions for future, broader, studies.

Having collected lexical data from the various sources listed above, we had to unify them in order to make them comparable with each other. This unification process, during which data from diverse sources are lifted to form a new, aggregated resource in which lexemes from different languages are aligned by their meaning and transcriptions are standardized to allow for phonetic comparison, can nowadays be done efficiently, thanks to new workflows and tools that have been proposed during the last decade.

In order to guarantee that we can compare translational equivalents for the lexemes in our sample, we mapped the French and English elicitation glosses in the original collections to the concept sets provided by the Concepticon project ( https://concepticon.clld.org, List *et al*., 2021b, Version 2.5). This procedure can be done quickly, because the Concepticon project now offers a variety of tools, including an automated mapping procedure for full concept lists, which can then be quickly manually refined, and a convenient lookup-tool for individual elicitation glosses in different languages ( https://digling.org/calc/concepticon, see List *et al.*, 2018).

In order to harmonize phonetic transcriptions provided in the different sources, we make use of orthography profiles ( Moran & Cysouw, 2018), which allow for a convenient conversion of graphemes (potentially consisting of more than one symbol) in one transcription system to graphemes in another. Orthography profiles are provided in a very straightforward tabular structure consisting of two basic columns, one representing graphemes in the source transcription and one representing the corresponding grapheme in the target transcription. Orthography profiles do not only allow us to convert text in one transcription system to text in another, they also allow us to simultaneously segment the transcription into meaningful units. For the purpose of lexical comparison in contact and historical linguistics, these units are distinct sounds in a given language. To guarantee that these sounds constitute meaningful units beyond our given data sample, we made sure that all distinct sounds in our sample can be linked to the Cross-Linguistic Transcription Systems reference catalog (CLTS, https://clts.clld.org, List *et al*., 2021a, Version 2.1), which offers references to more than 8000 speech sounds and allows for a convenient translation between different transcription systems (see Anderson *et al.*, 2018 for an overview on CLTS).

The data lifting was carried out with the help of the CLDFBench package ( https://github.com/cldf/cldfbench, Forkel & List, 2020), a software suite that allows us to combine the different stages of data lifting in a reproducible way, using the workflow that was established for the Lexibank repository of cross-linguistic wordlists (see List *et al.*, 2021c, https://github.com/lexibank/lexibank-analysed). To make sure that the data are comparable beyond the scope of a single application, CLDFBench essentially converts the data to the CLDF format, recommended by the Cross-Linguistic Data Formats initiative ( Forkel *et al*., 2018). For the specific analysis we employed in this study, we further converted our data to the tabular format required by the LingPy software package ( https://lingpy.org, List & Forkel, 2021, Version 2.6.9) and the EDICTOR interface ( https://digling.org/edictor/, List, 2021, Version 2.0, see List, 2017 for an overview). The supplementary material accompanying this study contains the data along with the CLDFBench Python code we used for data lifting. Table 2 provides a small example excerpt of our data to illustrate data lifting stages.

**Table 2.** From source to CLDF-compliant transcriptions and concepts.

Language	Group	Subgroup	Concept	Definition	Transcription	CLDF
Tiema Cewe	Mande	Soninke- Bozo	ANIMAL	animal (fr.)	ko̰o̰bo	k õː b o
Bunoge	Dogon	South- Western	ANIMAL	animal (eng.)	kɔ́mbɔ̀	k ⁵/ɔ ⁿb ¹/ɔ

***3.1.2 Genetic data.*** A total of 270 saliva samples were collected during a field trip in Mali under the permission of Malian and German ethic committees ( Babiker *et al.*, 2020). The samples were obtained from populations across the Bandiagara Escarpment and the sandy plains of central-eastern Mali. The generation of DNA samples and the downstream analysis were carried out, and are described by, Babiker *et al.* (2020).

### 3.2 Methods

***3.2.1 Linguistic analysis***

**A Automated identification of borrowing candidates**

Methods for the automated identification of cognates have largely increased in accuracy over the past decade (see List, 2014 for an introduction to basic techniques and List *et al.*, 2017 for an example on the comparison of different methods). While these techniques were primarily designed for the detection of genetically related words, scholars have repeatedly shown that they also can be used to identify borrowings (compare Van der Ark *et al*., 2007, and Mennecier *et al*., 2016, and see List, 2019 for an overview). In order to identify borrowing candidates automatically, it is advisable to use those cognate detection methods which are based on the identification of phonetic similarities among the languages in question, as opposed to those methods that try to identify deeper similarities based on automatically determined sound correspondences. For the purpose of identifying an initial set of borrowing candidates in our dataset of languages of central-eastern Mali, we use the Sound-Class-Based Phonetic Alignment algorithm (SCA, List, 2012), as it is provided as a method for automated cognate detection by the LingPy software package ( List & Forkel, 2021, Version 2.6.9). The SCA method starts by assembling pairwise distance scores derived from sound-class-based alignments for each word pair in a given concept slot across all languages in the sample and then uses a flat clustering algorithm to partition the words into sets of cognate candidates. In order to distinguish potential language-family-internal cognates from borrowings across families, we apply a filter that retains only those cognate candidates which occur in at least two different language families.

**B Manual refinement of automatically identified borrowing candidates**

**Figure 2.**
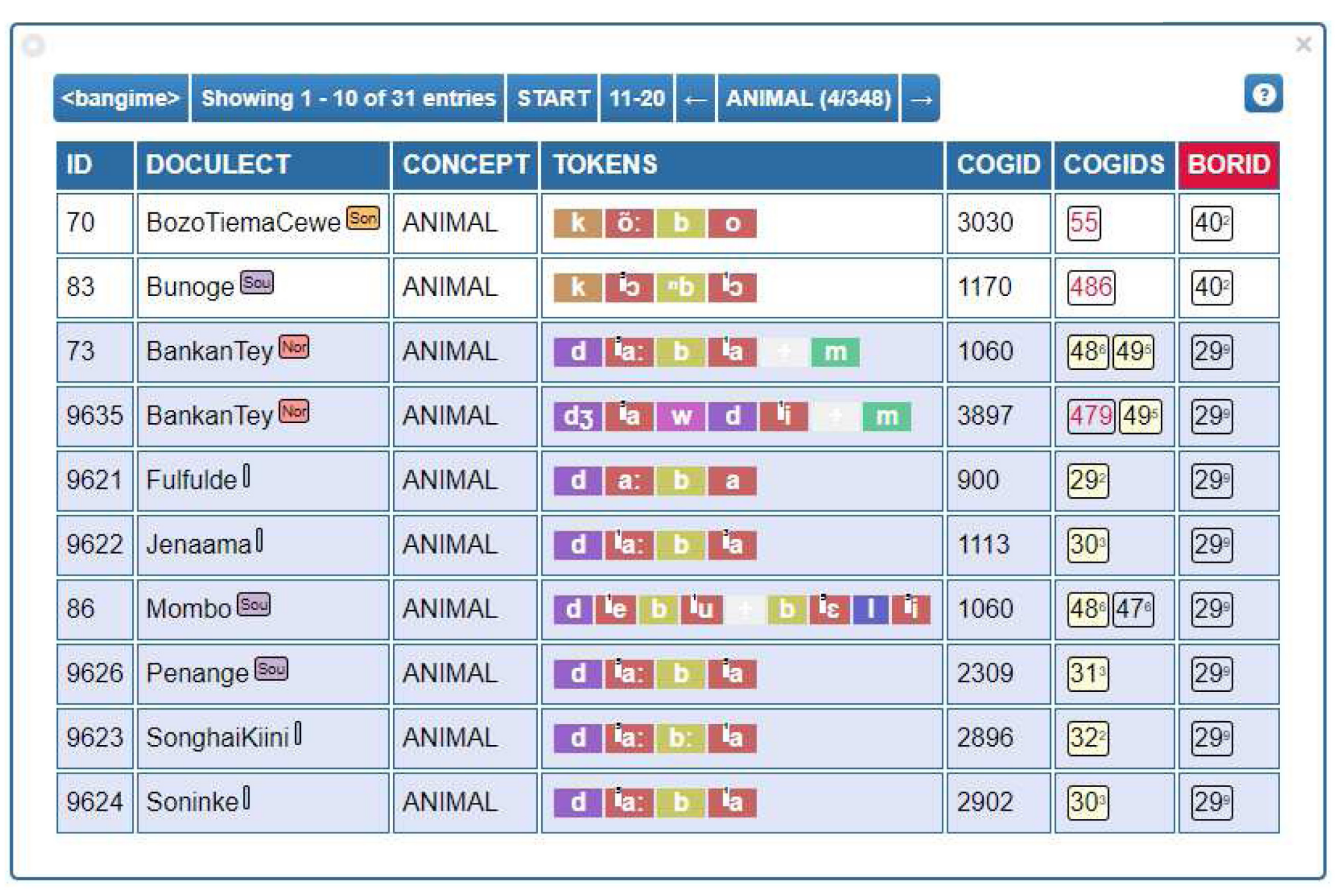
Cognates, partial cognates, and borrowing identifiers as visualized in the EDICTOR tool for the concept ANIMAL across some of the languages in our dataset.

Given that automated methods in historical linguistics still cannot compete with trained experts, we checked and refined the automatically identified sets of borrowing candidates manually. For this step of the analysis, we employed the Etymological Dictionary Editor (EDICTOR, https://digling.org/edictor/, List, 2021, Version 2.0), a web-based tool for the creation, curation, and annotation of etymological data in historical linguistics. The EDICTOR tool was written in JavaScript and offers an efficient framework for the annotation of cognate sets (see List, 2017 for an overview). Since our Bangime-based data do not consist of proper cognate sets, we slightly modified the traditional conventions for the annotation of borrowing candidates by providing zero identifiers for all those words that could not be identified as being shared across different language families, while using the typical numeric identifiers for cognate sets to assign words to the same sets of borrowing candidates. Figure 2 provides a screenshot illustrating how the data can be annotated with the help of the EDICTOR tool. For those interested in inspecting the database as it is presented in the EDICTOR tool, you can access the data *via* the link https://digling.org/links/bangime.html.

**C Loan distribution analysis**

Once putative borrowings across a larger set of languages have been identified, we can investigate these borrowing candidates in various ways. The most common approach in historical linguistics is to search for direct evidence for contact layers by investigating sound correspondence patterns of borrowing candidates. Typically, however, this analysis is restricted to language pairs, where the donor and recipient are well known (see, for example, the analysis by Lee & Sagart, 2008), given that sound correspondence patterns often turn out to be very complex, even when working with regular sound correspondences that do not result from borrowings. Additionally, whether evidence from sound correspondence patterns can be readily used in a stratification analysis, crucially depends on an array of factors, including the overall similarity of the languages with respect to their phonological systems, the intensity of contact, the specifics of loan adaptation, and the words involved in the borrowings themselves. As a result, the investigation of sound correspondence patterns often cannot be applied to the search for layers of contact. But when searching for potential hints on earlier contact scenarios, sound correspondences are not the only kind of evidence available to the linguist. First, semantic criteria may be used to check to which degree identified borrowing candidates belong to a coherent semantic field; we can take these “distributional properties of shared traits” ( List, 2019: 4) as evidence for a contact layer. Additionally, we can inspect the distribution of borrowing candidates across the languages in our sample. Assuming that language contact does not happen constantly, but rather in phases in the development of a language family, we can try to identify distributions that reflect past contact situations.

So far, in historical and contact linguistics, sufficient work has yet to be done with respect to the analysis of distributions of borrowings, and it is not entirely clear how large the dataset needs to be in order to be sufficient for valid quantitative approaches. Our goal is to provide a starting point for future studies on Bangime and its neighbors, be they quantitative, qualitative, or computer-assisted, thus it would be unrealistic to propose a full-fledged method for a loan distribution analysis here. We therefore apply a rather rough method for gaining first insights into the distribution of borrowing candidates in our data by investigating only those borrowing candidates which appear exclusively across two different language families. The basic idea is that—if enough of these examples can be found—borrowing candidates attested between family pairs alone could directly hint to past contact scenarios, while borrowing candidates covering several language families are much more difficult to analyze. This analysis itself can be carried out in a very straightforward manner: We first iterate over all sets of borrowing candidates in our data and then assemble statistics for all those candidates which cover only one language family. The results themselves are provided in the form of tables that can then be qualitatively inspected.

**D Implementation**

The methods described here are implemented in the form of Python scripts and are available along with the data and detailed instructions on the installation of required third-party libraries in the supplementary material accompanying this study.

***3.2.2 Genetic analysis.*** Babiker *et al*. (2020) investigated the genetic structure of populations across the Bandiagara Escarpment and the sandy plains of central-eastern Mali by genotyping ~600,000 SNPs for 210 individuals from 10 populations representing five ethnic groups (Bangande, Bozo, Dogon, Fula, and Songhai). The authors analyzed genome-wide data together with data from regional sets of populations and provided the detailed genetic structure of the populations from this region of West Africa ( Babiker *et al*., 2020).

## 4 Results

### 4.1 Results of the linguistic analyses

***4.1.1 General results.*** There are no direct borrowings (lexemes solely shared between two groups or languages) between Bangime and Fulfulde, the Songhai languages, or Soninke. Rather, Bangime directly shares vocabulary with Bambara, Jenaama (and its related Bozo languages in the sample), and the Dogon languages. This is not too surprising given that Fulfulde, Songhai, and Soninke are majority languages with wide reach, and that each of these is spoken in the immediate area of Bangime. On the other hand, direct borrowings between Bangime and Bambara are surprising given the geographic distance between the two groups. The proposed reason for this is discussed below. The following, Table 3, illustrates the number of concepts shared between groups. For the purposes of this general overview, we do not discuss the direction of borrowing.

In the following, we discuss the results of our computer-assisted methods to borrowing detection among the unrelated languages in our sample. To this end, we concentrate on Bangime. As a language isolate with no known relatives, it is impossible to find cognates, thus we must rely on borrowings as evidence for language contact. We have found that language, (also, a priori, speaker), contact is detected between not only geographically proximate, but also distant, languages. Furthermore, borrowings abound in Bangime in unexpected areas of the lexicon such as body parts and lower numerals. We present each of these findings in turn; rather than including lexical results in the text, we refer the reader to the supplemental materials.

**Table 3.** Shared concepts for each language family (out of 422 total concepts).

Group1	Group2	Total
Atlantic	Dogon	35
Atlantic	Isolate	4
Atlantic	Mande	12
Atlantic	Songhai	3
Dogon	Isolate	56
Dogon	Mande	111
Dogon	Songhai	45
Isolate	Mande	25
Isolate	Songhai	2
Mande	Songhai	10

***4.1.2 Specific results.*** Our analysis allows us to extract those cases in which language families share material exclusively between each other. We consider these examples particularly interesting, since they give us hints on specific situations of language contact, as these cases witness a much clearer shared past than we would find when investigating Wanderwörter in our data, whose origin is often difficult to trace. While all relations are potentially interesting, we exclusively concentrate on Bangime.

It would be beyond the scope of this paper to discuss all the results of this analysis in detail here, since more work and more discussions with colleagues will be needed to evaluate the findings properly and to re-check their consistency. For this reason, we will only provide an anecdotal discussion in which we show where we think the linguistic analysis of the presumed borrowings can help to shed light on the history of Bangime. Henceforth, we concentrate on the relation between Bangime and Dogon, Bangime and Mande, and Bangime and Songhai. These three language combinations were chosen because Bangime shares the most vocabulary with Dogon and Mande, but the least with Atlantic and Songhai; the latter two may be erroneous guesses or coincidence.

**A Bangime–Dogon (56 concepts)**

The most numerous borrowings into Bangime are, naturally given the projected identity of the Bangande as being and speaking Dogon, from Dogon languages. There are many words which are shared between Bangime and all the Dogon languages.

The Dogon languages form a tightly cohesive group. Many lexical items are shared among all the Dogon languages. Exceptions largely include borrowings from Songhai into the northern and eastern regions and from Mande languages into the western and southern areas. In terms of specific Dogon regions’ contact with Bangime, here, we examine the northeastern and southwestern Dogon areas.

**B Bangime–Northeastern Dogon**

The Dogon subgroup with which Bangime directly shares the most vocabulary is that which is spoken at the greatest geographic distance, not to mention the arduous terrain that separates the two peoples. The language with which Bangime shares the highest number of concepts is Bankan Tey. Furthermore, the majority of the 28 borrowed concepts shared with Bankan Tey have very low borrowing scores and high age scores in the World Loanword Database ( Haspelmath & Tadmor, 2009, https://wold.clld.org). Not only are these concepts numerous, one must note the prevalence of body parts and core vocabulary. Furthermore, honey cultivation from bees is essential to Bangande agricultural practices, yet the form for HONEY is shared.

**C Bangime–Southeastern Dogon**

Tommo So and Togo Kan, with 18 concepts each, are the languages of this group to share the most lexemes with Bangime. As with the northwestern group, most of these shared concepts are also found in other Dogon languages; MOON is an exception in being exclusively shared between Bangime and Tommo So. Given the discussion above in Section 2.5, that both the concepts SONG and SING are shared between Bangime and Tommo So is not surprising.

**D Bangime–Northwestern Dogon**

Most concepts shared between Bangime and the northwestern Dogon languages are also shared with the southwestern group, or with the Dogon languages more generally. Bondu So is the most prominent language in this group. For instance, Bangime shares lower numerals with Bondu So, which in turn patterns with the Dogon languages more generally.

**E Bangime–Southwestern Dogon**

Direct borrowings primarily from the southwestern Dogon languages are few; the languages of this group with which Bangime shares the highest number of concepts, 19 and 18, are its closest neighbors, Tiranige and Bunoge respectively. Furthermore, the few concepts not also shared with the northeastern and other Dogon regions such as DONKEY and WORK (LABOR) are additionally attested in the Mande language group.

Given the geographic proximity of the southwestern Dogon and Soninke–Bozo (discussed below) languages to Bangime, it is somewhat surprising that so few words are found in the Bangime lexicon directly from influence from these languages. In fact, that there are so few direct borrowings from these languages, and their prevalence among more recently introduced terms to the area, implies that the speakers’ contact has not been longstanding. On the other hand, both the Mande and Dogon languages are internally tightly knit, and thus finding words that do not have cognates with other languages of their families is rare. Furthermore, many words are shared among different groups of the area. Despite this, the tendencies remain striking.

**F Bangime–Mande (25)**

In terms of Mande groups, Bangime speakers today are in closest contact with Jenaama speakers; their language is one of the Bozo languages, which in turn is most closely related to Soninke. However, the Mande language with which Bangime shares the most vocabulary is Bambara.

**G Bangime–Bambara**

Among the 15 concepts shared between Bangime and Bambara, seven can be considered to be associated with the caste system of the Mali Empire, and more could be added if other language groups were also included such as HOST which is also attested in Fulfulde. For instance, the term for BLACKSMITH in Bangime is a direct borrowing from Bambara, not shared with either the any other Mande, or Dogon, languages.

Although two concepts are also found in Jenaama, none are shared with Soninke, and thus purportedly are not from the earlier Ghana Empire with which the Soninke language was associated. One notable exception to this generalization is the concept SLAVE, which is likely borrowed from Soninke into Bangime, but not directly, as it is also found among the southwestern Dogon languages, and not in Bambara.

Additionally, note that the majority of the words shared between Bangime and Bambara include body parts, and even those from the caste system have low borrowability scores and high age scores; the lexeme for the concept GOD is not that of the widely distributed Muslim term Allah, rather the pre-Muslim name for God, *ŋara* ~ *ŋala*.

**H Bangime–Jenaama**

Over half, seven out of a total of 13, concepts shared between Bangime and Jenaama are also shared with Bambara. Even if we factor in concepts shared between Bangime, Jenaama, and the neighboring Dogon languages, the numbers remain quite low in comparison to those shown above for Bangime and the Dogon languages which are spoken at greater distances. 

**I Bangime–Soninke**

Only two concepts, EAR and TRAP (PITFALL), are borrowed into Bangime directly from Soninke. However, it does not seem as if neither of these are coincidental. Note that ELEPHANT, a compound of the concepts BIG and EAR is also shared between Bangime, Jenaama, the Bozo languages, and, somewhat surprisingly, the Dogon language Bondu So. The form for TRAP (PITFALL) is probably the result of a slight semantic shift. The form ɡɛŋɡɛ for IRON is found in both the Dogon language Gourou and Bangime, which in turn, as noted above, is likely a borrowing from Bambara.

**J Bangime–Bozo**

There are only two lexemes which are found in Bangime and the Bozo languages as a group, but exclude Jenaama and Bambara: the form for HOT in Bangime is identical, save for tones, to that of three Bozo languages. However, it is possible that the form is actually borrowed from Dogon languages of the northeastern group, which are (albeit distantly) related to the Mande languages, and thus the phonetic similarities in Bangime are somewhat of a coincidence. On the other hand, MILLET is a concept that is essential to the livelihood of all the cultures in the sample, and thus the most common form is shared between all Dogon languages save for the southwestern group and Mande languages Bambara and two Bozo languages; the form found in Bangime and the Bozo languages in which it is attested, is an outlier.

**K Bangime–Songhai (2)**

Save for two concepts, ANCESTORS and SEVEN, which could be attributed to chance, there are no Bangime-Songhai pairings in which other languages are not also implicated, illustrating that the chances of direct contact between Bangime and Songhai are low. An interesting trend, however, is the fact that the two Dogon languages that have most impacted Bangime, Bankan Tey and Bondu So, are those that have the most shared concepts with Songhai. Geographically, the areas where Bankan Tey and Bondu So are spoken are the closest among the Dogon groups to where Songhai Kiini and Songhai Senni are spoken. Thus, shared concepts among those areas are not surprising.

**L Bangime–Atlantic (4)**

Today, Fulfulde speakers have frequent contact with those of Bangime, and nearly all Bangande are fluent in Fulfulde, but there are hardly any direct loans between them that exclude the Dogon languages. This implies that, as long as Bangime has been in contact with Fulfulde speakers, so have Dogon.

**M Dogon–Mande (111)**

In principle, at least, Dogon and Mande are related at the higher order of the Niger-Congo language phylum. This is reflected, whether by contact or a common inheritance, by extensive shared vocabulary in our dataset. Phonotactically, the difference is difficult to distinguish as sound correspondences can be found in both cases. Semantically, certain concepts such as BOAT are likely a recent introduction into plains Dogon languages from Bozo fisherman, whereas the origin of concepts such as MARROW, with variable forms and pairings within the subgroups, is less clear.

Synonyms and semantic extensions are a crucial component of any study of borrowed lexical items. Borrowings are common among our wordlist when a concept is expressed through more than one word. An extended meaning of the concept HEAD is an areal feature found throughout all the groups in our sample except Mande. For instance, HEAD as *kṵṵ* is shared between Bambara and many eastern Dogon languages with which speaker contact is limited today. However, this is one of two forms found in the Dogon languages; the other is *dana*. If the language in question lists both forms, only that which is shared with Bambara is used metaphorically for a reflexive object, ‘on top’ and in compounds such as ‘chief’, whereas the *dana* Dogon form is used exclusively to refer to the body part. As the Dogon form for HEAD which refers only to the body part is not shared with any other group, it is likely the native term while the one shared with Bambara is borrowed. Bambara, in turn, does not share the form *kṵṵ* with any other Mande language in our sample. Perhaps the form was borrowed at an early stage from the Dogon languages into Bambara but without the extended meaning.

**O Dogon–Songhai (45)**

As noted above, Bondu So and Bankan Tey speakers have the most contact today with those of Songhai. Otherwise, although most Dogon languages share the word for BLACKSMITH with Songhai, the term [ *dʒɛ́mɛ̀-nɛ̀ / dʒɛ́mɛ̀-m*] more likely is originally derived from the Dogon word for ‘black’ [ *dʒɛ́m*]. In this case, our results reflect both the diachronic as well as the synchronic situation. This is discussed further below in the following sections.

### 4.2 Results of the genetics analyses

Patterns of genetic diversity in the Bandiagara cliffs of central-eastern Mali reflect complex population dynamics and deep history of settlements in this region of the West African fringe. The results show that the populations of central-eastern Mali have strong affinities to West Africans, in particular, Niger–Congo speaking West Africans. Interestingly, the genetic variation is driven, at some level, by the linguistic diversity and subsistence patterns. 

**Figure 3.**
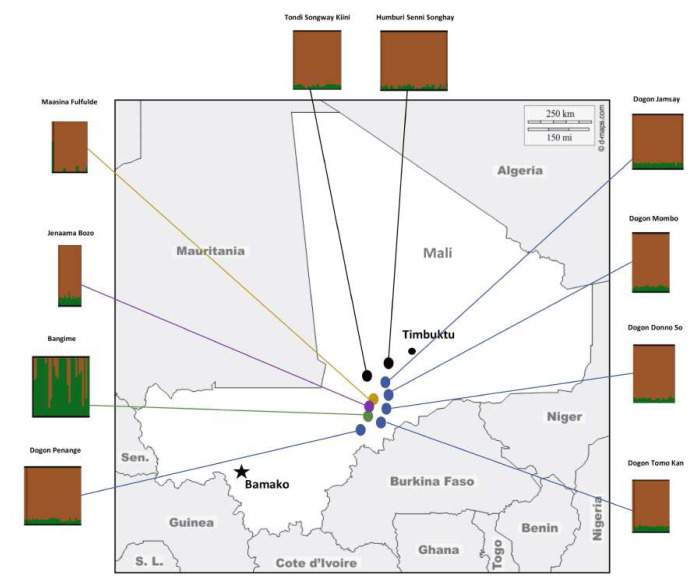
Map of the Bandiagara Escarpment in central-eastern Mali showing the locations and the genetic structure of populations studied. Figure adapted with permission from ( Babiker *et al*., 2020).

***4.2.1 Bangande.*** The Bangande population is genetically distant from the surrounding Dogon, Bozo, Fula and Songhai populations ( Figure 3). The unique Bangande genetic structure and the high levels of homozygosity compared to the populations from central-eastern Mali are probably the result of a long-term isolation ( Babiker *et al*., 2020). Although the genetic structure of the Bangande is distinct from all other populations in the region, Babiker *et al*. (2020) reported some admixed individuals whose genealogical records point to an origin from a non-Bangande ethnic group where the Dogon languages are spoken ( *e.g*., Tommo So). Even though it is not common to report cases of marriage between the Bangande and the neighboring populations ( Heath & Hantgan, 2018), the data of the genealogical records show that women (mostly grandmothers of the studied participants) were brought to the Bangande villages from other Dogon villages, *e.g*., Tiranige, Tommo So, and Penange. Among these individuals, some were blacksmith participants in the Bangande caste system (see Section 2.6). Also, the study inferred a long-term effective population size for the Bangande population (Ne = 3,276 (CI 2,947–3,711)), which is the smallest in comparison to the other studied populations. Moreover, the mean time of divergence between the Bangande and other populations from central-eastern Mali was estimated to be 9,900 (CI 8,726–10,838) years ago. The estimated time of divergence for the Bangande is in line with the “time barrier” of the conventional linguistic comparative methodology ( Gray, 2005) and suggests a time depth between language families. These findings suggest that the Bangande population is a genetic isolate that has resisted assimilation and language replacement despite the people’s claims that they are Dogon. Also, the findings suggest that the Bangande might represent one of the earliest inhabitants of the region prior to the Dogon expansion and linguistic diversification.

***4.2.2 Dogon.*** The five Dogon populations studied in Babiker *et al*. (2020) are representative of three additional Dogon linguistic groups based on areas relative to the cliff range: Escarpment Dogon, West Dogon, and Plains Dogon ( Hammarström *et al*., 2021). The genetic structure of the Dogon groups shows homogeneity at smaller K clusters K=2 (best cross-validation error for the Admixture analysis) ( Figure 3). However, the populations show some level of structure at K cluster (see Babiker *et al*., 2020), possibly reflecting the differences in levels of inbreeding within each group rather than different genetic ancestries. The study also showed smaller pairwise genetic distances (FST) between the Dogon Donno So (Escarpment Dogon) and the Dogon Mombo (West Dogon) populations as well as between the Dogon Jamsay and Tomo Kan (Plains Dogon). Furthermore, the genetic data allowed the estimation of population divergence times, which pointed to an older divergence for the Dogon of the cliffs. In detail, the study reported a divergence between the Mombo and Penange (West Dogon) linguistic groups at (3,031 (CI 2,723–3,301) years ago and between the Dogon Mombo (West Dogon) and Donno So (Escarpment Dogon) linguistic groups at (2,373 (CI 2,080–2,673) years ago. In contrast, the study reported a relatively recent divergence between the Dogon Plains groups: the Dogon Jamsay and Tomo Kan linguistic groups at (1,059 (CI 908–1,104)) years ago.

***4.2.3 Bozo.*** The millet-farming Bozo studied by Babiker *et al*. (2020), are distinguished from other fishing Bozo of this region and inhabit a village on the cliffs of the roadside that extends along the valley that leads to the Bangande villages. Although the Bozo population is geographically closer to the Bangande than other populations in the study ( Figure 3), it clusters with the Dogon populations in the ADMIXTURE analysis and bears low pairwise genetic distances from other populations in comparison to the high distance from the Bangande.

***4.2.4 Fulani.*** The Fulani population of the Bandiagara Escarpment represents a distinct level of genetic diversity mostly driven by gene flow from Eurasian/admixed African populations (see Figure 3 and Babiker *et al*., 2020). The subsistence pattern and the nomadic nature of the Fulani might have eased the channels of language contact with other groups in the region, but not genetic relationships through marriage, which are controlled by different social and political aspects.

***4.2.5 Songhai.*** Further, Babiker *et al*. (2020) showed a contrast in genetic structure between the two Songhai linguistic groups studied ( Figure 3), the Songhai of Humbori, the speakers of Humburi Senni and the Songhai of Kikara, the speakers of the Tondi Songway Kiini. While the analysis pointed to a Dogon influence on the genetic structure of the Songhai of Kikara (limited gene flow), it showed that the genetic structure of the Songhai of Humbori was influenced by gene flow from non-African sources. The study highlighted the political role of Humbori during the Songhai polities and Empire dating from the 7/8 ^th^ to 16 ^th^ centuries AD and the possible connections with other populations that was also facilitated by the geographical location of the town.

## 5 Discussion and conclusion

### 5.1 Proposed layers of language contact

Speakers of Bangime and Bambara are in infrequent direct contact today, and yet shared vocabulary items are found between the two languages to the exclusion of the others in the sample. The closest Bambara speakers are found in cosmopolitan areas such as the regional capital Mopti where Bangande travel occasionally for work or business interests. Otherwise, the country’s capital city Bamako is quickly becoming a frequent destination for Bangande seeking salaried jobs. Naturally, migrants return to their home in the Bandiagara Escarpment, but these trajectories mostly affect young people and our data were gathered from consultants who had spent the majority of their lives in the village and environs. Additionally, we can see a clear theme, especially among the direct borrowings from Bambara into Bangime. Granted, Bambara is the only west Mande language in our sample, and thus these forms likely have Mande cognates among languages of that sub-grouping. However, it is worth noting that many concepts are shared between Bangime and Bambara to the exclusion of related Mande languages or surrounding Dogon languages.

With few exceptions, concepts are clearly related to the caste system and, thus likely, the Mali Empire. The only notable omission to this generalization is the concept SLAVE which is found in Bangime as well as the surrounding southwestern Dogon languages and is from Soninke, but is not shared with Bambara. Concepts shared among Bangime, Jenaama and the closest neighboring Dogon languages appear to be relatively recent. Furthermore, Bangime has no direct borrowings from Soninke. Thus, it is proposed that the Bangande formed part of the Mali Empire before their inclusion with the Dogon community, between 1200–1600 AD. The Dogon populations, in turn, were also impacted by Bambara speakers as part of their empire, but in a different location than the Bangande. As the Mali Empire was huge, there is no way to tell as of yet where the Bangande lived. It is unlikely that the Bangande were a part of the Ghana or the Songhai Empire.

### 5.2 Language contact through population dominance

As stated by Sands (2019), there are not many studies that have examined the effects of past kingdoms and empires on the languages of Africa. Tamari (1991) is a notable exception, who highlights the fact that loanwords abound throughout cultural vocabulary used to designate roles in the caste system. Without regular sound correspondences indicated language change, the introduction of most lexical borrowings is notoriously difficult to date, however, by pairing lexical data with that of historical records and genetic findings sampled from populations in the area, we propose the following timeline for language contact between the groups in our sample.

Evidence of pearl millet cultivation around 300 kilometers north of the Bandiagara Escarpment is among the earliest attested in West Africa, dating back to the 4th millennium BC ( Burgarella *et al*., 2018; Manning *et al*., 2011). Yet, the form for MILLET is unique to those who currently inhabit the southwestern quadrant of the Bandiagara Escarpment, implying that they likely cultivated millet prior to other Dogon groups, but following the split therein. Bangande perhaps had early contact with Bozo speakers today located around Lake Debo. On the other hand, the form for MILLET found in Bambara is widespread, including the other Dogon groups, and thus could have been spread later through the Mali Empire, yet this would mean that, apart from the southwestern quadrant, Dogon did not practice early millet-growing, perhaps because they lived too far south, and therefore in too-wet of a climate for domesticated millet crops to thrive.

In further support of this hypothesis, it would seem as if Proto-Dogon had no word for HORSE or CAMEL (both of these are clearly borrowed), which implies they lived south of the tse-tse fly belt (albeit which was further north than it is today ( *c.f.* Steverding, 2008), thus it could have been at their current location which is now drier than before). It can be said that horses were introduced to Dogon-speaking peoples from Bambara-speakers after the split from Bondu So-speakers who obtained the term from Songhai-speaking invaders. Camels were introduced to Bankan and Ben Tey-speaking populations by Songhai speakers after they split from other Dogon-speaking populations; southwestern Dogon and Bangime had contact with Soninke speakers at a time when slaves became a part of their caste system, but before their respective contact with other Dogon groups.

Additionally, the term for PIG was borrowed from Songhai into the southwestern Dogon languages suggesting that these languages were in contact with each other prior to arriving at their current location (that is, prior to having contact with either Bangime or Jenaama). Additionally, this is another case of lexical replacement, but it is slightly complex. In each of the languages, PIG also means warthog. Thus, the form for concept PIG is that which is used among the Eastern Dogon languages, along with Yorno So and Yanda Dom (geographically Southeastern languages which pattern genetically with the northwestern group) is the ‘native’ term, although it is likely a Gur borrowing from languages outside of our sample. The form from Songhai, however, is solely used for the concept PIG; warthog is a separate lexeme. Thus, in the case of the Eastern Dogon languages, PIG was borrowed from Songhai with the same meaning, but then was expanded to encompass warthog as well.

Songhai still influences adjacent Bondu So and Bankan Tey speakers more than any others. Blench (2015) argues that, “there was once a branch of Nilo-Saharan, now submerged, spoken on the Bandiagara” (ibid: 74). Pertinent to our study, he uses comparative Bangime-Dogon-Songhai lexical data to support his claim of a lost Nilo–Saharan substrate which he calls ‘Plateau’ as he believes there is evidence for traces of this lost language among the cliff-dwellers today. Of the 12 lexemes he provides, over half are monosyllabic, thus increasing the likelihood of chance resemblances. While certain others are somewhat convincing, such as NOSE, Hantgan and List (under review) have discussed the similarities between Bangime body parts and those of Dogon but with mixed meanings; see NOSE in Bangime in comparison with EAR among many of the Dogon languages. Others such as CLOUD and RIVER are considered in our sample to be borrowings, rather than cognates, as Blench suggests for the former (albeit he admits the latter). On the other hand, he states that there are lexical resemblances between the Dogon languages and Songhai which exclude Bangime. Of these, the only one we deem plausible is HORN (ANATOMY); however, this form is most certainly shared with Bangime as well as Dogon and the Songhai languages and is thus a relatively recent relic of language contact among the groups.

### 5.3 Genetics is key to understanding patterns of linguistic diversity

The genome-wide genetic data provides details about the genetic landscape of populations from central-eastern Mali and reveals the mystery of Bangime language isolate and its speakers. Furthermore, the study reports limited admixture in the Dogon, the Bozo and the Songhai of Kikara pointing to the use of the cliffs as a refuge and a barrier to genetic mixtures. These recent results of the Dogon update previous interpretations of the Dogon structure ( Tishkoff *et al*., 2009). In contrast, it shows evidence of admixture signals in the nomadic Fula and the Songhai of Hombori, which we interpret in the context of gene–culture interactions for the Fula. This study has implications for whether the Bangande are genetically descended from the ancient Bandiagara people collectively known as the Pre-Dogon, and for whether its language was spoken before the Dogon Expansion.

This work highlights the importance of interdisciplinary research to resolve the contentious paradox of language isolate classification and linguistic diversity in Africa. The recent findings are important for promoting multidisciplinary research including genetics, archaeology, historical linguistics and anthropology. It shows the importance of interdisciplinary research in answering big questions in the respective disciplines and to a larger extent in human history. Further, it allows engaging the population history in an area with complex linguistics and genetic history and linking demographic changes to historic events.

The depiction of genetic diversity in West Africa is critical for reconstructing West African demographic history and modern human origins in Africa. In addition, this genetic information has the potential to reveal genetic relationships among distant populations and together with linguistic data will be informative for understanding the co-evolution of genes and languages. Furthermore, multidisciplinary research will help to depict the complex linguistic and genetic histories, hence, decoding unanswered questions in human history.

## 5.4 Conclusion

Sands (2019: 8) states, “There are no surveys of language contact for the majority of Niger-Congo and Benue-Congo subgroups”. She notes that among Niger-Congo subgroups that have been studied in Sahelian West Africa, most involve Gur languages contact with groups such as Mande and Kwa. Furthermore, Gur languages have been discussed to have had contact with Songhai languages of the Nilo-Saharan branch ( Souag, 2012), and Songhai-Mande contacts have been discussed for some time ( *c.f.* Creissels, 1981). Ours is the first study to examine the effects of language contact at the lexical level among such a wide sample of sub-groupings, crossing phyla boundaries. Further, it is the first that displays a congruence between a language isolate and the genetic structure of its speakers in West Africa. Moreover, the deep divergence of the Bangande ~ 9,900ya from the surrounding population in the region supports the hypothesis that the Bangande represents the earliest Bandiagara people ( Heath & Hantgan, 2018) and provides insights into the region’s past.

## Data availability

### Underlying data

Zenodo: underlying data for ‘First steps towards the detection of contact layers in Bangime: a multi-disciplinary, computer-assisted approach’. https://doi.org/10.5281/zenodo.5751226

This project contains the following underlying data: the raw data used for the study before and after it was processed using the computer-assisted methods outlined above, the sources of the original data, the scripts used to process the data, and the borrowing plus cognate patterns found in the data.

## Software availability

Source code available from: https://github.com/lexibank/baf2

Archived source code at time of publication: https://doi.org/10.5281/zenodo.5751226

License: Creative Commons Attribution 4.0 International license (CC-BY 4.0).
