# Supplementary material for: First steps towards the detection of contact layers in Bangime: a multi-disciplinary, computer-assisted approach
Source: Open Res Eur. 2022 Apr 22;2:10. Originally published 2022 Jan 21. [Version 2] doi: 10.12688/openreseurope.14339.2 (PMC10445931; doi:10.12688/openreseurope.14339.2)
Supplement: Peer review history

# Peer review: Reviewer response for version 1

**DOI:** 10.21956/openreseurope.15476.r28437

**Published:** 2022-03-23

**Authors:** Rebecca Grollemund

**Affiliations:** 1 Department of English, University of Missouri, Columbia, MO, USA

This paper entitled “ *First steps towards the detection of contact layers in Bangime: a multi-disciplinary, computer-assisted approach*” aims at studying Bangime, an isolate language spoken in Central-Eastern Mali by using novel computer-assisted technologies in order to better understand the history of the Bangande (speakers of the Bangime).

 As an isolate language spoken in a region where we can observe a high linguistic diversity with languages belonging to different language families (Niger-Congo and Nilo-Saharan), the status and the (linguistic) classification of Bangime remain unclear. Bangime however does share some lexical items (basic vocabulary) with surrounding languages such as Dogon or Fulfulde, revealing therefore that the Bangande have been in contact with the speakers of the neighboring languages. In order to sort out the status of Bangime, the authors propose a multidisciplinary approach combining linguistic and genetic data. Regarding the linguistic data, the authors focused on the study of the putative borrowings found between Bangime and the surrounding languages in order to better understand the layers of contacts. The authors are also using computer-assisted techniques to help them identify these putative borrowings.

 I have found this paper very interesting for several reasons. First, it is true that Bangime is a fascinating language because of its particular status: it is an isolate surrounded by languages belonging to different language families. Therefore, trying to recover parts of its history by studying loanwords in order to better understand the ancient connections between Bangime and the other languages is very exciting.

 Second, the methods (computer-assisted techniques) used for this paper are also very novel and cutting-edge. Usually, in historical linguistics, when we want to identify loanwords, we have to apply the Comparative Method that will allow us to extract regular sound correspondences for the languages studied. We have to have a good knowledge of the languages studied in order to find the phonological clues that are going to point to the loanwords. In this paper, the authors are using tools developed for automated identification of cognate sets which are based on the study of phonetic similarities. In order to identify loanwords (and not cognate sets) with these automated methods, the authors applied an additional filter that has identified cognate words that occur in at least two different language families. For example, when they found a word in Bangime that was cognate with Dogon and Mande, this word would be assumed to be a loanword. The results they have obtained and presented in Table 3 are very interesting. The high number of cognate sets shared between Mande and Dogon languages makes sense because they belong to the same Niger-Congo family (this number reflects common ancestry, shared retentions). But then, the (relatively high) number of cognate sets shared between Bangime and Dogon languages does not reveal common ancestry (because they are not related) but contacts and therefore borrowing! In section 4.1.2., the authors proposed a review of the loanwords shared between Bangime and the other languages. My only regret here is that they do not present concrete examples (such as in Figure 2). Maybe they could have an annexe in which all the examples would be presented because I would be very interested in studying these loanwords.

 Finally, in order to better understand the history of the Bangande, the authors combined their linguistic findings with genetic findings and proposed a new hypothesis regarding the chronology and the layers of language contact.

 I recommend this paper pass peer review. The paper is very clear and well written. I think the idea of using lexical borrowing in order to investigate the layers of contact for Bangime with their neighbors was very interesting. Because Bangime is an isolate language, we do not have many options if we want to better understand its history.  

 I have just small remarks/questions that need to be addressed:

 First, in section 3.1.1, the authors wrote that the languages studied in Table 1 belong to “4 languages families” where the correct number is two because Mande, Dogon and Atlantic languages belong to the Niger-Congo family.)

 If the authors are only using cognate sets (shared between more than two language families), why did they take the time to align all the data? Were they able to spot some interesting phonological patterns?

 If I am not mistaken, “borrowing” corresponds to the linguistic process whereas “loanwords” correspond to the loanwords identified.

If applicable, is the statistical analysis and its interpretation appropriate?

Yes

Is the study design appropriate and is the work technically sound?

Yes

Is the work clearly and accurately presented and does it engage with the current literature?

Yes

Are the conclusions drawn adequately supported by the results?

Yes

Are sufficient details of methods and analysis provided to allow replication by others?

Yes

Are all the source data and materials underlying the results available?

Yes

Reviewer Expertise:

African languages with a focus on Bantu and Bantoid languages, historical linguistics with a focus on linguistic classification, phylogenetic methods and language evolution

I confirm that I have read this submission and believe that I have an appropriate level of expertise to confirm that it is of an acceptable scientific standard.

# Response

**Published:** 2022-04-09

**Authors:** Abbie Hantgan-Sonko

**Affiliations:** LLACAN, France

The authors appreciate the insightful comments of this reviewer. We have provided answers to the questions posed in the text as follows (reviewer comments in italics):

*My only regret here is that they do not present concrete examples (such as in Figure 2). Maybe they could have an annexe in which all the examples would be presented because I would be very interested in studying these loanwords.*

 AUTHORS ADDED: “We have clarified now that all examples can be easily accessed via the supplementary material and that we focus here on major patterns.” We have also added one example per pattern in the inline text.

*First, in section 3.1.1, the authors wrote that the languages studied in Table 1 belong to “4 languages families” where the correct number is two because Mande, Dogon and Atlantic languages belong to the Niger-Congo family.)*

 AUTHORS CHANGED: 4 to 3, but with the note that our groupings are based on current classifications in Ethnologue as well as Glottolog.

*If I am not mistaken, “borrowing” corresponds to the linguistic process whereas “loanwords” correspond to the loanwords identified.*

 AUTHORS ADDED: “In some traditions, there is a distinction between loanwords and foreign words, pointing to different time depths of integration. We decided to use "borrowing" as a neutral term throughout the text.”

*If the authors are only using cognate sets (shared between more than two language families), why did they take the time to align all the data? Were they able to spot some interesting phonological patterns?*

 AUTHORS ADDED: “Alignments are only used for word pairs in order to assess similarity scores between words and can be visualized later with the help of the EDICTOR tool to make sure that identified borrowings show enough overall similarity. We have clarified this in the main text, adding one sentence specifying that the automated alignments are used for word-pair distance calculation.”

# Peer review: Reviewer response for version 1

**DOI:** 10.21956/openreseurope.15476.r28471

**Published:** 2022-02-22

**Authors:** Laura McPherson

**Affiliations:** 1 Department of Linguistics, Dartmouth University, Hanover, NH, USA

This paper lays out a methodology for drawing together genealogical, archeological, and linguistic evidence to reconstruct settlement history. In doing so, it offers a preliminary analysis of Bangime, a language isolate spoken in central Mali whose speakers (the Bangande) claim to be ethnically Dogon but who are genetically and linguistically distinct. Understanding the settlement patterns for language isolates is challenging because there are no linguistic relatives for comparative/reconstruction data, but the authors show how identifying likely borrowed terms from surrounding languages and coupling this with genetic and archealogical evidence can help us understand patterns of contact and settlement in the absence of a written record.

 Overall, I found the paper very interesting and well written. The authors did a good job of acknowledging the limitations of the study while at the same time focusing on methodology so that others can replicate, expand upon, or argue against the analysis. This kind of interdisciplinary work offers exciting new avenues into understanding the prehistory of many regions of the world that lack a written record.

 I have a few relatively minor comments and suggestions which I lay out below page by page:

- Last sentence of plain language summary: “computational linguistic” -> “computational linguistics”
- Second to last paragraph of Section 1: What does “pre-ethnic” mean?
- Last sentence of Section 1: Remove comma after “follow up on”
- First paragraph of Section 1.1: “while Songhai is that of Nilo-Saharan” -> Maybe, “while Songhai is a branch of Nilo-Saharan”?
- Last paragraph of Section 1.1 could be fleshed out more. Non-linguistically savvy readers may not know what cognates mean and why we can’t rely on them in the case of a language isolate.
- First sentence of Section 1.3.1: Something about the parenthetical about art historians come across as strange. Think about reworking it.
- Middle of 1.3.2, “the puzzle of pieces” is a strange collocation.
- Last paragraph of 1.3.2 is a really nice summary of the paper as a whole.
- Section 2.1: “Among our Mande groups, Bambara, Bozo, and Soninke, …” Perhaps offset the three groups from the rest of the sentence either with parentheses or m-dashes. The commas made the sentence hard to follow.
- Last paragraph of Section 2.2: “Our samples from the Kikara, ….” this sentence is also really hard to follow, since it isn’t immediately clear that Tondi Songwai Kiini is related to Kikara, and that Humburi Senni is related to Humbori. Please rework.
- Section 3.2.1 B, this section could be fleshed out and explained more clearly. I was a little lost on the methods.
- Section 4.1.1, beginning: Make it more explicit here that there is loads of borrowing between Fulfulde and multiple language families, just not direct borrowings, as you define them here.
- Section 4.1.2 E, regarding DONKEY and WORK, if they are shared by Mande, wouldn’t this make them not direct borrowings? The last paragraph of this section could also be clarified a bit.
- Section 4.1.2 I, “ However, it does not seem as if neither of these are coincidental.” Too many negatives here.
- Section 4.1.2 M, there are cognates for kṵṵ in many other Mande languages, see Vydrin “On the problem of the Proto-Mande homeland”
- Section 4.2.2, extra open parenthesis before the years at the end of the paragraph.
- First sentence of 4.2.3, remove comma after “studied by Babiker”
- Section 4.2.5, missing comma after “the speakers of Humburi Senni”
- Section 5.2, first paragraph, “indicated” -> “indicating”. Also, the comma before “however” should either be a semicolon or a period.
- Section 5.2, third paragraph, spell out why the tse-tse fly belt is important to camels and horses.
- Section 5.2, last paragraph, “suggests for former (albeit admits the latter)” What does this mean?
- Section 5.3, something is wrong in the first sentence, “Bangime language isolate” Also, “these recent results of the Dogon”… the Dogon what?
- Section 5.4, missing apostrophe (?) in “Gur languages contact”

If applicable, is the statistical analysis and its interpretation appropriate?

Yes

Is the study design appropriate and is the work technically sound?

Yes

Is the work clearly and accurately presented and does it engage with the current literature?

Yes

Are the conclusions drawn adequately supported by the results?

Yes

Are sufficient details of methods and analysis provided to allow replication by others?

Yes

Are all the source data and materials underlying the results available?

Yes

Reviewer Expertise:

I am a linguist with expertise in the geographic/linguistic areas under study. I cannot comment on the genetic or computational approaches.

I confirm that I have read this submission and believe that I have an appropriate level of expertise to confirm that it is of an acceptable scientific standard.

# Response

**Published:** 2022-04-09

**Authors:** Abbie Hantgan-Sonko

**Affiliations:** LLACAN, France

R1: Section 4.1.2 M, there are cognates for kṵṵ in many other Mande languages, see Vydrin “On the problem of the Proto-Mande homeland”

 AUTHORS ADDED: "...among many Mande languages (as also exemplified by Vydrin (2009: 131)".

# Response

**Published:** 2022-04-09

**Authors:** Abbie Hantgan-Sonko

**Affiliations:** LLACAN, France

The authors are grateful to this reviewer's careful overview of our submission. We have made all the corrections suggested by the reviewer (indicated in itlaics), as well as added additional commentary, detailed as follows:

*Second to last paragraph of Section 1: What does “pre-ethnic” mean?*

 AUTHORS ADDED: “ Bandiagara Escarpment which is currently home to the Bangande and neighboring populations was occupied by peoples that  potentially pre-dated these ethnic delineations known throughout the literature simply as the ‘Tellem’; we leave this matter to future research.”

*Last paragraph of Section 1.1 could be fleshed out more. Non-linguistically savvy readers may not know what cognates mean and why we can’t rely on them in the case of a language isolate.*

 AUTHORS ADDED: “Cognates, following List (2016), are etymologically related words, stemming from a common proto-form, which explicitly ignores borrowings. Regarding of the use of the term, “borrowing”, in some traditions, there is a distinction between loanwords and foreign words, pointing to different time depths of integration. We decided to use “borrowing” as a neutral term throughout the text.”

*Section 2.1: “Among our Mande groups, Bambara, Bozo, and Soninke, …” Perhaps offset the three groups from the rest of the sentence either with parentheses or m-dashes. The commas made the sentence hard to follow.*

 AUTHORS ADDED: “However, among these, Jenaama, the Bozo population in this study, is not necessarily representative of the main Bozo groups of fishing villages along the Niger River and its floodplains.”

*Last paragraph of Section 2.2: “Our samples from the Kikara, ….” this sentence is also really hard to follow, since it isn’t immediately clear that Tondi Songwai Kiini is related to Kikara, and that Humburi Senni is related to Humbori. Please rework.*

 AUTHORS ADDED:”We collected samples in Kikara from the speakers of Tondi Songwai Kiini (hereafter simply ‘Kiini’), which is distinguished from other Songhai languages as the “mountain Songhay language” (Heath, 2005a). Further, we collected samples in Hombori, where Humburi Senni Songhay (which we refer to as ‘Senni’) is spoken. Both languages belong to the eastern division of the Songhai languages spoken in Mali, which have been classified within the Nilo-Saharan language superfamily.”

*Section 3.2.1 B, this section could be fleshed out and explained more clearly. I was a little lost on the methods.*

 AUTHORS CLARIFIED:  We added additional explanation of the manual refinement procedure by adding a longer and more detailed caption to Figure 2.

 AUTHORS ADDED: “That is, after the automatic methods assigned numbers to cognate sets as well as to borrowing sets, we manually inspected each set for accuracy and changed any spurious sets.”

*Section 4.1.1, beginning: Make it more explicit here that there is loads of borrowing between Fulfulde and multiple language families, just not direct borrowings, as you define them here.*

 AUTHORS ADDED: “In our sample, there are no direct borrowings (lexemes solely shared between two groups or languages) between Bangime and Fulfulde, the Songhai languages, or Soninke. That is, while languages such as Fulfulde have impacted all of the languages in the region, the borrowing patterns between Bangime and Fulfulde also encompass the Dogon languages.”

*Section 4.1.2 E, regarding DONKEY and WORK, if they are shared by Mande, wouldn’t this make them not direct borrowings? The last paragraph of this section could also be clarified a bit.*

 AUTHORS CLARIFIED: This is a good point to which I returned to the data in order to add examples for both lexemes. I also added: “Our methods did not detect these words as being shared with Mande languages though the forms are close. Further inspection of these patterns may lead to more generalized patterns.”

*Section 4.1.2 M, there are cognates for kṵṵ in many other Mande languages, see Vydrin “On the problem of the Proto-Mande homeland”*

 AUTHORS ADDED: “...among many Mande languages (as also exemplified by Vydrin (2009: 131)”.
